# The fed-batch principle for the molecular biology lab: controlled nutrient diets in ready-made media improve production of recombinant proteins in *Escherichia coli*

**DOI:** 10.1186/s12934-016-0513-8

**Published:** 2016-06-17

**Authors:** Mirja Krause, Antje Neubauer, Peter Neubauer

**Affiliations:** Laboratory of Bioprocess Engineering, Department of Biotechnology, Chair of Bioprocess Engineering, Technische Universität Berlin, Ackerstr. 76, ACK 24, 13355 Berlin, Germany; Laboratory of Developmental Biology, Faculty of Biochemistry and Molecular Medicine, Biocenter Oulu, University of Oulu, Aapistie 5A, 90220 Oulu, Finland; BioSilta Europe GmbH, Berlin, Germany

**Keywords:** Recombinant protein production, EnBase, Shake flask, High cell density, Expression

## Abstract

While the nutrient limited fed-batch technology is the standard of the cultivation of microorganisms and production of heterologous proteins in industry, despite its advantages in view of metabolic control and high cell density growth, shaken batch cultures are still the standard for protein production and expression screening in molecular biology and biochemistry laboratories. This is due to the difficulty and expenses to apply a controlled continuous glucose feed to shaken cultures. New ready-made growth media, e.g. by biocatalytic release of glucose from a polymer, offer a simple solution for the application of the fed-batch principle in shaken plate and flask cultures. Their wider use has shown that the controlled diet not only provides a solution to obtain significantly higher cell yields, but also in many cases folding of the target protein is improved by the applied lower growth rates; i.e. final volumetric yields for the active protein can be a multiple of what is obtained in complex medium cultures. The combination of the conventional optimization approaches with new and easy applicable growth systems has revolutionized recombinant protein production in *Escherichia coli* in view of product yield, culture robustness as well as significantly increased cell densities. This technical development establishes the basis for successful miniaturization and parallelization which is now an important tool for synthetic biology and protein engineering approaches. This review provides an overview of the recent developments, results and applications of advanced growth systems which use a controlled glucose release as substrate supply.

## Background

For many reasons *Escherichia coli* is still the preferred choice as a host system for protein production. With relatively low costs one can achieve high biomass and high protein yield in only short cultivation times. Furthermore, *E. coli* is extremely well-studied in its biochemical and physiological characteristics. With a wealth of tools available *E. coli* also can be easily adapted as needed by genetic manipulation. However, even though the general procedure for protein production is straightforward, protein aggregation during expression is still a major obstacle. Different approaches are commonly applied to address this problem, and to optimize protein folding while maximizing protein expression. The currently available expression systems with their advantages and pitfalls have been regularly reviewed [1–4]. A smart combination of the different parts of the system (e.g. prokaryotic or eukaryotic host organism, type of plasmid with its specific features) can lead to an improved expression. Additional conventional approaches for protein expression optimization are the coexpression of chaperons, use of codon optimized genes, alternate protein tags, change of cultivation medium, production process optimization [2, 5]. The choice of the system influences the success of proper protein folding and hence the production of active, soluble protein. Even more specialized systems facing folding problems have been developed. The pre-expression of Erv1p sulfhydryl oxidase and disulfide bond isomerases for example is a robust technique for the production of disulfide bonds containing proteins [6, 7].

Since every protein is different, the expression and purification strategies must be defined for each single case. In their review, Gräslund et al. [2] summarized that there are many choices to make when expressing proteins regarding all the parts of the system; e.g. selection of *E. coli* strain, the fusion of the protein with a His-tag or another tag, the application of a T7 RNA polymerase expression system or another regulated promoter system, and finally the choice of the medium and cultivation conditions. They published a consensus protocol which they agreed to be a good starting-point when aiming to produce a recombinant protein. Nevertheless, success is protein dependent and a robust and ever-working strategy is still missing. They pointed out that the choice of the growth strategy has a significant influence on the success of protein expression. A major concern is the direct correlation of the degree of aeration and the cultivation conditions such as temperature and medium used, with the expression level and the solubility of a recombinant protein [2]. However, this is rarely considered in molecular laboratories even though one is clearly aware of this fact in the field of biotechnology and bioprocess. During recent years, finally the direction of approach has changed. Possible solutions offered, tried to address the problem via optimizing the cultivation medium. One of these developments for high-level protein production is the autoinduction system [8], which works with the T7-RNA polymerase based pET plasmids and other isopropyl beta-D-thiogalactopyranoside (IPTG)-inducible bacterial expression systems under the control of *lac* operon regulatory elements. In the first growth phase *E. coli* consumes the preferred carbon substrate glucose until depletion before the diauxic shift to lactose consumption induces the protein expression. Additionally, the cells start to use glycerol as a second major carbon source which is available in the system.

**Fig. 1:**
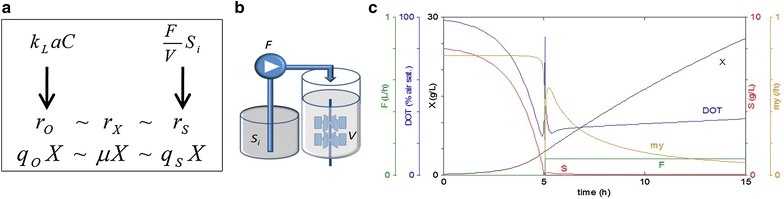
Overview of the fed-batch principle. **a** The oxygen transfer capacity k_L_aC in a bioreactor limits the volumetric oxygen consumption rate r_O_ if aerobic conditions need to be maintained. Due to the relationship between the volumetric rates for growth (r_X_), substrate consumption (r_S_) and oxygen (r_O_), r_O_ can be controlled by a control of the volumetric substrate uptake by a controlled substrate feed (*F* Feed rate, *V* reactor volume, *Si* substrate concentration in the feed solution). As the volumetric rates are the product of the specific rates (q_O_, μ, q_S_) with the actual biomass X, at a constant feed rate the specific rates decrease at increasing biomass and thus the level of dissolved oxygen is dependent on the feed rate, but largely independent on the cell density. **b** Principle view of a fed-batch bioreactor with a feed reservoir and the fee pump which controls the fee rate. **c** A typical simulation plot for a fed-batch cultivation on mineral salt medium with glucose as the only carbon source and a constant feed rate. The *graph* shows the Feed rate (F), dissolved oxygen tension (DOT), biomass (X), Substrate (S, often glucose) and the specific growth rate (my)

However, an autoinduction system does not necessarily provide any means of control in terms of cultivation conditions and hence the metabolic state of the production strain. Unlimited availability of nutrients and exponential growth in batch type systems lead to the production of side-metabolites like acetate and the subsequent acidification of the cultures [9]. On the contrary, the deamination of amino acid substrates, which derive from the utilization of peptones or yeast extract as a carbon source, can cause an increase in pH (since excess ammonia is secreted into the medium) [10]. Both processes are a major reason for flask-to-flask variations [11, 12]. The rate of supply of the different nutrients in combination with the oxygen availability influence cell growth and protein expression substantially. The balance of all metabolic rates is a major issue for well controlled and robust expression—a challenge due to changing specific amino acid composition of the protein, which affects directly the cellular pools of all distinct amino acids and due to the sensitive regulation of the pathways of synthesis and transport of the amino acids also global responses. The issue of controlled cell physiology and process robustness, i.e. limitation of batch-to-batch variation, is well considered in industrial bioprocesses where glucose based mineral salt media are normally used and complex additives are avoided [13, 14]. Control of the cell metabolism is obtained by continuous feed of the only carbon source in a way that the feeding rate limits the uptake rate. This nutrient limited fed-batch technology is the dominating technology in the bioindustry to reach high cell density fermentations. The feed rate of a highly concentrated nutrient solution, which is glucose in most cases, directly affects the growth rate and the rate of respiration. As the metabolic flux through the glycolysis is closely related to the respiratory activity by the reduction of NAD^+^ to NADH + H^+^, the glucose feed rate is directly linked to the consumption of oxygen. Oxygen supply is always a limitation in aerobic processes due to the limited solubility of oxygen in aqueous media. During batch cultivations in shaken cultures where all available substrate is added from the start of the cultivation, optimal oxygen levels require 10–20 % culture volume in relation to the flask volume [15]. A controlled carbon source (usually glucose) supply is a key way to control the volumetric oxygen consumption rate. Therefore, the fed-batch cultivation where the carbon source is added continuously is the preferred alternative to a batch cultivation, as metabolic rates can be controlled [16]. If the volumetric oxygen consumption rate is smaller than the volumetric oxygen transfer rate, the culture experiences aerobic conditions independent on cell density, although the specific growth rate (i.e. the growth rate of a single cell) is lower the higher the cell density is (cf. Fig. 1a). It is however clear that at a higher volumetric oxygen transfer rate, the glucose consumption rate and thus the final cell density is higher compared to cultures with a low volumetric oxygen transfer rate. High cell densities can therefore be reached if a high oxygen transfer rate is enabled and a proper control of the glucose flux is guaranteed [16].

**Fig. 2:**
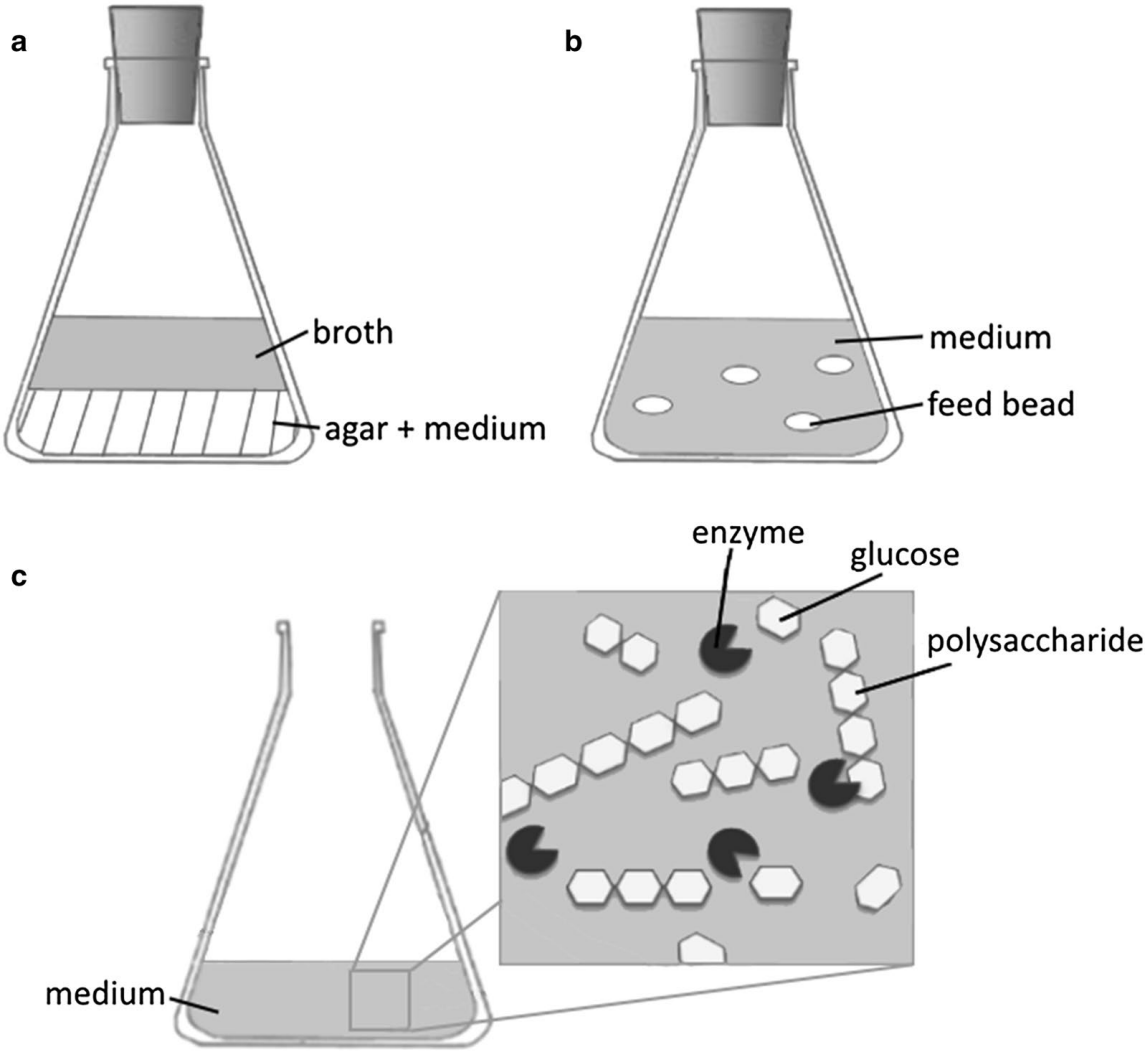
Overview of cultivation systems with integrated substrate supply. **a** Multilayer system by [17, 21]. A medium-agar was covered by a broth in which the bacteria were cultivated. **b** Feed Bead^®^ System by Jeude et al. [19]. Silicone elastomer discs (feed beads) releasing substrate to the medium. **c** Principle of the integrated substrate delivery system with the EnBase technology [11]. Dissolved polysaccharides being part of the cultivation medium are degraded to glucose molecules by the addition of defined enzyme concentration

While in industry mechanical pumps regulate the substrate feed rate to maintain a certain limitation of the carbon source (see Fig. 1b, c), it remained a challenge to apply the fed-batch technology in smaller laboratory cultivation systems, such as shake flasks and microwell plates. Since a few years pump-independent intelligent growth systems with internally controlled substrate release are available and make the fed-batch technology attainable for small scale cultivation. The idea of an integrated nutrient supply system is not new and has been first published as early as 1958. An overview of integrated feed systems can be found in Fig. 2. In 1958 Tyrell et al. [17] used a two phase system composed of a nutrient agar covered by liquid growth medium (Fig. 2a). Later this system was adapted to Streptomyces cultures providing controlled ammonia feed [18]. Recently, the described principles were transferred to intelligent growth systems available for small scale cultivation in different formats. One example is the Feed Bead^®^ technology [19] where silicone elastomer discs containing glucose crystals are added to the cultivation and release the substrate by diffusion (Fig. 2b). The only way to control substrate release is implemented by the amount of beads added to the culture. Based on the same principle of diffusion, Wilming and colleagues [20] developed a special microwell plate. Here the feeding solution reaches the wells with cultivations through a capillary system filled with a hydrogel. The substrate diffuses through the hydrogel into the culture well, and the feed rate can be adjusted by changes in the geometry of the channel and the substrate concentration gradient. In contrast to this, the *EnBase*^*®*^ cultivation technology does not rely on substrate diffusion, but involves a possibility to control the feed rate without the need for an external pumping system [11, 21]. Instead, a soluble polysaccharide is degraded by a biocatalyst, and thus glucose is gradually released into the culture as the primary carbon source (Fig. 2c). The amount and activity of the biocatalyst directly controls the release rate of glucose over the time. This technology was specifically optimized for recombinant protein production by considering the following points: firstly, a constant delivery of glucose until the end of the protein production phase. Secondly, a self-sustainable pH by the addition of a balanced mixture of inorganic and organic ammonia compounds during glucose limitation. Thirdly, a possibility to adapt the system to different aeration conditions by adjustment of the amount of enzyme added [11, 21]. Different studies convincingly show that growth and protein production are not necessarily connected, but that a limitation of the dissolved oxygen during the production phase even may be advantageous [22–24].

Furthermore, the system has been optimized for ease of applicability and is available as pre-sterilized tablets which contain the full medium. These tablets are simply added to sterile water and dissolve in the first minutes of cultivation. Studies have shown that the technology can be easily combined with conventional approaches to yield not only higher amount of a target protein through the higher cell density [21, 25, 26], but also in many cases a higher amount of correctly folded protein per cell [26, 27]. Modern screening systems, such as RAMOS, Biolector and PreSens’ SensorDish with an ability for on-line monitoring of growth data provide excellent tools to optimize the growth and expression conditions [28–32].

This positive effect is obtained due to lowered growth rate, which in turn is related to a lowered rate of protein synthesis and an improved balance between the synthesis and the folding rates. The current list of successful expression examples with EnBase and similar continuous release techniques (see Table 1) indicate, that controlled growth systems are not only the solution if current standard cultivation methods do not succeed but become a standard approach. This review aims to expand the researchers’ current view on recombinant protein production towards technologies which became recently available (see Table 2). These technologies promise to further advance protein production strategies improving protein quality and yield alike.

**Table 1:** Overview of produced target molecules with advanced growth system

Target group	Specific target	References
Enzymes	Monomeric variants of *Trypanosoma brucei* triosephosphate isomerase(TIM)	[21]
	*Drosophila melanogaster* multifunctional enzyme type 2 (MFE2)	[11]
	A-domain of human protein disulphide isomerase (PDI)	[11]
	*Lactobacillus* alcohol dehydrogenase (ADH)	[11]
	Tryptophan synthase complex	[52]
	Formate dehydrogenase (FDH) from *Candida boidinii*	[93]
	Human NSUN4, 5-methylcytosine RNA methyltransferase	[34]
	SdrE protein—surface adhesin of *Staphylococcus aureus*	[35]
	Zmp1—zinc-dependent metalloprotease	[36]
	Human lipoxygenases hALOX15	[38]
	Esterase, Est 22; Axe A and Axe B	[44, 54]
	*Rhizopus oryzae* lipase (ROL)	[84]
	Purine nucleoside phosphorylases (PNPs)	[26, 103–105]
	Alkaline phosphatase (PhoA) and a phytase, AppA	[58]
	Surface lipoprotein SitA from *Staphylococcus pseudointermedius*	[42]
	Endopolygalacturonase from *Aspergillus niger*	[32]
	Chloramphenicol acetyltransferase I (CATI)	[45]
	Alkane monooxygenase (AlkB)	[43]
	Pseudomonas aeruginosa lipoxygenase (PA-LOX)	[39]
	Sortases: SrtC1-2b, Srt2-2b, SrtA	[46]
	Nitrilases	[51]
	Rat lactate dehydrogenase	[53]
	Zebrafish ALOX2 (lipoxygenase)	[40]
	Zinc‑dependent metalloprotease Zmp1	[101]
	α-amylase AmyM from *Geobacillus stearothermophilus*	[88]
	*Rhizopus oryzae* lipase (ROL) in *P. pastoris*	[84]
Antibodies, antibody fragments, other binding proteins	Two single-domain antibodies (sdAbs)	[68]
	Four Fab fragments	[24]
	Anti-interleukin 1ß scFv	[58]
	Zinc transporter 8 autoantibodies (ZnT8A)	[49]
	Different nanobody variants	[60]
	10 scFv and 10 Fab fragments	[70]
	DNA binding protein LiaR	[41]
	DNA binding transcriptional regulator NadR (Neisseria adhesin A regulator)	[100]
	RNA-binding proteins: nucleolin EF-hand domain-containing protein D2 (EFHD2), splicing factor U2AF (U2AF653) serine/arginine-rich splicing factor 1 (SRSF1)	[47]
	Fully reduced active RNase inhibitor: contains leucine-rich repeat motifs (LRR)	[22, 48, 98]
Disulfide bonded proteins^a^	Reteplase, a fragment of tissue plasminogen activator which contains only the kringle 2 and protease domains (nine disulfide bonds)	[7]
	Chimeric-truncated form of tissue-type plasminogen activator (t-PA)	[33]
	Human leukemia inhibitory factor (hLIF)—a cytokine (three intramolecular disulfide bonds)	[50]
	AppA, a phytase (four disulfide bonds)	[58]
	Human growth hormone hGH	[58]
	Human-derived glycosyltransferase GalNAcT2 (five disulfide bonds)	[59]
Bioactive peptides	Super-large non-ribosomal peptide synthetase valinomycin synthetase subunits Vlm1 (370 kDa) and Vlm2 (284 kDa)	[72]
	Nonribosomal peptide valinomycin	[73, 75, 106]
	Polyketide macrolide 6-deoxyerythronolide B (6dEB) in *Bacillus subtilis*	[76]
	Ribosomal peptide microviridin	[71]
Others	Plasmid DNA	[77–79]
	Capripoxvirus proteins 64 and 95	[25]
	Fusion protein: multiple-epitope antigen CTB-UE	[69]
	Saporin L3 from *Saponaria officinalis* in *Pichia pastoris*	[81]
	Human immunoglobulin receptors in *P. pastoris* (extracellularly)	[83]

^a^Antibodies and antibody fragments are mentioned in a separate section

**Table 2:** Commercially available intelligent growth media for the production of recombinant proteins

Working principle	Product name	Company	Application
EnBase^®^ technology: controlled release through enzymatic degradation of polymers	EnPresso^®^ B	BioSilta Ltd	Protein production with *E. coli*
	EnPresso^®^ Y Defined	BioSilta Ltd	Protein production with yeasts
	EnPresso^®^ B Defined Nitrogen-free	BioSilta Ltd	Production of ^15^N-labelled proteins in *E. coli*
Autoinduction technology	Overnight Express™ Autoinduction System 1	Merck-Millipore Novagen	Lac-promoter based protein production with *E. coli*
	MagicMedia™	Thermo Fisher Scientific	Production of recombinant proteins in *E. coli* with T7 RNA polymerase system
Slow-release polymer systems for substrates, including glucose	FeedBeads^®^	Kuhner Shaker	Microbial growth under substrate controlled (limited) conditions
	FeedPlate^®^	PS—Biotech	Microbial growth under substrate controlled (limited) conditions
Unknown principle	FIFTYOD	Prozomix	Production of recombinant proteins in *E. coli * BL21

### Application of advanced growth systems in *E. coli* cultivations

Controlled metabolism by a fed-batch type of operation incorporated into microbial growth systems like the EnBase technology comprise several benefits such as aerobic metabolism to high cell densities and lower production of acidifying side products and thus a stable pH over long cultivation times. Also the growth at a constant volumetric growth rate, i.e. approximately linear increase of OD, is an advantage compared to exponentially growing cultures in view of robustness and reproducibility. As a result, the final product yield is less independent on the time or cell density of induction [11, 21]. For example, Peck et al. [25] observed eight times higher cell densities and an increased protein yield per cell producing capripoxyvirus proteins which causes severe diseases in sheep and cattle. The production of Tissue-type plasminogen activators (t-PA) is commonly done in mammalian cell cultures. However, one faces problems like low yields, long cultivation times and high costs. Mahboudi et al. [33] has successfully overcome these problems by producing a biologically active truncated form of the tissue plasminogen activator in *E. coli BL21 (DE3)* using the EnBase technology. They obtained an 8 to 20-fold increase in cell density and an improved specific activity. Growth systems that can offer controlled cultivation conditions can be used as an easy tool for the production of a variety of proteins in *E. coli*—usually *E. coli* BL21 (DE3). For example, the multifunctional enzyme 2 from Drosophila [27] and the human MTERF4-NSUN4 protein complex that regulates mitochondrial ribosome biogenesis [34] enabling crystallization of both proteins, Sdr proteins from *Stapholococcus areus* [35], a zinc-dependent metalloprotease Zmp1 from *Clostridium difficile* [36], human proteins such as different arachidonate lipoxygenase enzymes [37, 38], lipoxygenase from *Pseudomonas aeruginosa* [39] and zebrafish [40], the cytosolic response regulator *LiaR* from *Enterococcus faecalis* expressed in the pETDuet vector [41], the metal-dependent surface lipoprotein *SitA* from *Staphylococcus pseudointermedius* [42] or another metalloenzyme like the alkane monooxygenase AlkB [43] were successfully produced employing the EnBase technology. Furthermore, a family VIII esterase Est22 [44], the *E. coli* chloramphenicol acetyltransferase I (CATI) [45], different sortase enzymes from *Streptococcus* [46], and proteins which do not express in commonly applied media like the phosphoprotein nucleolin [47] and eukaryotic ribonuclease inhibitor [22, 48] could be successfully expressed in *E. coli* by the application of an advanced growth system. To allow a tunable expression by variation of the activity of the T7 RNA polymerase, enzyme based substrate supply media can be combined with Lemo21 *E. coli* (DE3). In the Lemo System the expression of the natural inhibitor of T7 RNA polymerase, the T7 lysozyme (*lysY*), is controlled by the rhamnose promoter. The amount of rhamnose controls the activity of the T7 RNA polymerase and is chosen at a concentration to minimize the cellular stress, which has been useful for the functional expression of membrane proteins and other difficult to express targets. One example is the expression of specific zinc transporter 8 autoantibodies (ZnT8A) important in diabetes [49].

Another example for successful combination of different optimization strategies was the functional production of the human leukemia inhibitory factor (LIF): codon-optimization for high expression, application of Origami B (DE3) with thioredoxin coexpression for correct disulfide bond formation and Profinity eXact™ for simpler purification was combined with the EnBase technology for high cell density cultivation [50]. The soluble rhLIF yield was estimated to be about 1 mg g^−1^ of wet weight cells, with a purity of >98 %. In addition, a study reporting the discovery of new nitrilases for dinitriles describes the combination of *E. coli* Origami B (DE3), arabinose induced co-expression of GroEL/ES chaperones, low IPTG concentration, expression temperature of 25 °C and the EnBase technology as the most efficient method after optimization of several cultivation parameters [51]. Another study [52] combined a pET vector system and the commercial *E. coli* strain BL21-CodonPlus (DE3)-RIPL which expresses additional copies of certain t-RNA genes (*argU*, *ileY*, *leuW)* with EnBase. For this protein the final yield was increased by a factor of 16 compared to LB (Luria Bertani) medium. A further study applied *E. coli* OverExpress C41 BL21(DE3) cells with a reduced activity of the T7 RNA polymerase and a higher tolerance membrane and other toxic proteins. These cells were used to produce tetrameric rat lactate dehydrogenase in 50 ml EnPresso growth system at the same level as obtained with 1 L traditional LB medium [53]. The authors succeeded with the purified protein to obtain the crystal structure. Also Mokoena et al. [54] were able to express two carboxyl ester hydrolases identified in a metagenomic study in EnPresso medium. These enzymes can de-acetylate cephalosporins which are valuable building blocks in the production of semi-synthetic β-lactam antibiotics. The production was possible, because an intelligent growth system was employed. In a bigger study Achenbach et al. expressed a large set of proteins for in vitro protein synthesis with EnPresso medium [55].

#### Disulfide bond containing proteins

The correct formation of disulfide bonds, a major post-translational modification, is important for the production of many proteins, in particular secreted mammalian proteins like hormones, growth factors and immunoglobulins [56, 57]. One common approach is the secretion of such proteins to the periplasm in *E. coli* to ensure a favorable folding environment [1]. This approach can be combined with a growth system providing the controlled cultivation conditions as was successfully demonstrated by Matos et al. [58] for a four disulfide bond containing protein and by Nguyen et al. [7] for a nine disulfide bond containing protein (vtPA). In the latter case significant improvements in active protein yield (800 times), with a 100-fold increase in activity and in cell density (10 times) could be achieved. A very recent example is the expression in *E. coli* of functional recombinant human glycosyltransferase GalNAcT2 with five disulfide bonds [59]. The protein sequence was optimized towards expression in *E. coli* and a plasmid was co-expressed which encodes the sulfhydryl oxidase Erv1p and the protein disulfide isomerase PDI facilitating improved formation of disulfide bonds. This set-up was combined with the application of *E. coli* SHuffle^®^ strain and EnPresso B cultivation medium. Due to the reproducible conditions provided by the medium, it was possible to scale-up the whole expression system to the 1.5 L fermentation scale. Furthermore, the protocol to produce nanobodies in *E. coli* SHuffle^®^ showed an enhancement from 22 to 45 mg L^−1^ when cells were cultured with the advanced growth system instead of LB [60].

#### Autoinduction

It is well known that lactose can replace IPTG as potent and cheap inducer for recombinant protein production [8, 61]. This has been realized in autoinduction media (e.g. ZYM autoinduction medium from Novagen) which provide the benefit that culture does not need to be interrupted for induction, which is a big advantage in large screening campaigns but also in view of the time scheduling of shake flask experiments. By autoinduction the protein is expressed automatically by derepression of the P_lac_-derived promoter variant when glucose becomes exhausted, due to the well-known diauxic behavior which has been originally described in the PhD thesis by Jacques Monod [62]. This diauxic regulation is a result of two concurrent events, glucose catabolite repression and inducer exclusion. Studier [8] describes that by using autoinduction media, the induction is smooth and typical drawbacks like strong formation of inclusion bodies are a lower problem, possibly due to slower growth rate and lower expression of the effective T7 RNA polymerase. From understanding of the diauxic adaptation [63] one might even assume that induction of the stress responses during the transient from glucose to lactose consumption lead to this improved robustness. While it is known that lactose can be also applied in combination with glucose limited fed-batch cultivations [64], recently Ukkonen et al. [23] have shown that glucose limitation is important rather than glucose starvation. Protein expression after the diauxic switch is working well with a continuous background feeding of glucose. If this background feeding of glucose is adjusted properly, lactose only serves as an inducer, but is not catabolized. In a paper by Mayer et al. [65] the concentration of lactose stayed approximately constant during a 24 h cultivation with a background enzymatic supply of glucose while the product was smoothly produced in a correctly folded form at reasonable high cell densities (OD_600_ of >20).

#### Oxygen

In common shake-flask cultivations the availability of oxygen is usually problematic. And even if the oxygen availability and subsequently the oxygen transfer rates can be improved, the commonly applied media cannot facilitate the growth to very high cell densities. However, feed-controlled systems can balance this through constant substrate limitation and hence provide robust cultivation conditions even at higher oxygen transfer rates. This was shown by Ukkonen et al. [66] and also by Pilarek et al. [67] in two different approaches. Ukkonen et al. [66] combined the enzyme based substrate delivery technique with Ultra Yield Flasks (Thomson Instruments) characterized by four-fold increased oxygen transfer. While standard media like LB or TB (Terrific Broth) lead to a high amount of insoluble protein, the application of the feed-based system enables higher biomass production (up to 20 g L^−1^) and higher enzyme activity (>100-fold improvement). Pilarek et al. [67] provided additional oxygen to their miniaturized cultures by the addition of perfluorodecalin (PFD) which can be charged with oxygen and will release the oxygen during cultivations while taking up CO_2_. In combination with advanced growth systems this lead to a 40 % increase in the OD_600_ of *E. coli* cultures. Usually perfluorocarbons are applied in mammalian cell cultivations, but as demonstrated can also be used in bacterial cultivations.

#### Peptides, antibodies and antibody fragments

Immunoglobulins are applied widely in therapeutic and diagnostic applications. The yield of correctly folded, functional antibody fragments, such as Fab-fragments, is usually rather low. The addition of a signal sequence to the Fab-fragment sequence directs the Fab-fragments to the periplasm when expressed in *E. coli*. Leakage of folded fragments into the medium can occur and facilitates easy purification. Studies showed that the application of an optimized expression system with an intelligent growth technology such as EnBase increases the amount of total and also extracellular yields [24]. Zarschler et al. [68] were able to increase the yield of functional and soluble signal domain antibodies of up to 200 mg L^−1^. In another study, the expression level of a vaccine epitope (CTB-UE) which can induce cellular and humoral immune responses and attenuate *Heliobacter pylori* infections could be increased by almost 15 % with a solubility rate of about 90 % [69]. Recently, the CyDisCo system was introduced which enables production of disulfide bonded proteins in the cytoplasm with reducing pathways by coexpression of a catalyst of disulfide bond formation and a catalyst of disulfide bond isomerization [6, 7]. Gaciarz et al. [70] show the efficient formation of ten natively folded and active scFvs with a yield of up to 240 mg L^−1^ and 10 Fab antibody fragments up to 42 mg L^−1^ by using jointly the CyDisCo and EnBase technologies. The use of intelligent growth medium clearly outperformed standard media like TB or LB.

Also the production of translationally modified bioactive ribsosomal peptides seems to benefit from a growth control. The yield of novel microviridin variants in *E. coli* was improved by using EnPresso B medium directly from fosmids with the modifications and proof of their biological activity [71].

#### Non-ribosomal peptides

Also the production of non-ribosomal peptides is challenging due to the need to functionally express the superlarge multidomain nonribosomal peptide synthetase which often consist of more subunits, the need to express the phosphopantetheinyltransferase for the activation of the thiolation domains and eventually additional factors, and finally to keep the cell alive to drive the synthesis of the wanted peptide. As an example we have recently expressed successfully the nonribosomal peptide and macrolactone antibiotic valinomycin. Its production was heavily increased by switching from TB to the EnBase cultivation system [72–74]. The fed-batch process is advantageous for the production of such complicated systems as a stable metabolic state can be maintained over a longer period of time [73, 74]. Large proteins are very often prone to misfolding and aggregation, but the selection of a fine-tuned cultivation system comprising of a weak promoter, low induction conditions and controlled growth conditions made the production of the peptide with two separate domains, encoded as separate genes (domain 1 is 370 kDa, domain 2 is 284 kDa) possible, and the final yield of valinomycin was even comparable to the productivity of several native Streptomyces strains [72, 75]. A 33-fold increase, leading to 10 mg L^−1^ of product was obtained. Additionally, the NRPS was used to produce the final product, the antibiotic valinomycin. Such processes usually require a quite long cultivation time (>50 h), and hence cannot work in a batch cultivation environment. It is interesting to remark, that the EnBase technology also was the solution for the production of the polyketide 6-deoxyerythronolide with *B. subtilis.* This PKS multienzyme complex was expressed under control of the acetoin induced *acoA* promoter [76].

### Other applications of advanced growth systems

Furthermore, studies and the development of new protocols show that intelligent growth systems are not only beneficial when applied for the production of proteins or peptides, but can also increase the yield of isolated plasmid [77]. The production of gene-libraries is often limited to the amount of plasmid produced. Additionally, a large number of samples needs to be handled in parallel and requires a robotic set-up to enable automated handling. This on the other hand demands downscaling of the culture volume. It would be of great benefit to achieve a high optical density in a small cultivation volume. Pilarek et al. [78] and Grunzel et al. [77] showed that it is possible to miniaturize *E. coli* cultures while obtaining a high final yield in plasmid when advanced growth systems are applied controlling the cultivation conditions. Ramirez et al. [79] concluded in a study that the use of their engineered strain together with the enhanced growth system is a promising method for plasmid DNA production in shake flasks. This demonstrates once more that when exploiting bacteria as production factories in general, the cultivation conditions are [5] of great importance, and should be considered as an essential part during any optimization studies.

For protein production in yeasts the same challenges as in *E. coli* cultures apply. The application of an advanced growth systems in yeast cultivations has been successfully tested by Grimm et al. [5]. The growth and the ability to convert a certain substrate of twenty different yeast strains was compared in the study. In almost all cases the cell mass (measured as wet cell weight) and the substrate conversion (in %) was significantly improved compared to cultivations done in standard YEP medium. Potvin et al. [80] proposed the application of intelligent growth systems in *Pichia pastoris* cultivations. Recently, studies have demonstrated the feasibility of this approach. Yuan et al. [81] produced the type 1 ribosome inactivating protein Saporin L3 from *Saponaria officinalis* (soapwort) leaves which is toxic for *E. coli* successfully in *P. pastoris* using the EnBase technology. Ruth et al. [82] successfully applied the Feed Bead^®^ Technology to study transcription factor of *P. pastoris* and Ashoor et al. [83] to produce human immunoglobulin receptors with EnPresso in *P. pastoris*. In the approach by Panula-Perälä et al. [84] the slow glucose release of the EnBase technology was used as a continuous background energy and carbon supply bridging the gaps between methanol pulses needed for protein expression under the AOX1 promotor. Such a background feeding provided a significant increase in cell densities and product activities.

Recently, Grimm and Neubauer [85] published that the combination of such growth systems can be easily combined with on-line sensor plates, e.g. for oxygen or pH. This enables a controlled cultivation where not only pH, OD and temperature can be followed and controlled, but also the substrate availability. Therefore, the cultivation runs under optimized, robust and reproducible conditions reaching higher cell densities. All these examples show that application of advanced growth systems is not limited to *E. coli*. Such systems even have been used with Bacilli [86] which is interesting, since Bacilli have native amylases which can interfere with the growth system. However as amylases are expressed only under nutrient limitation, even with Bacilli an initial growth control is possible (own non-published experiments). A better control is obtained if *amyE* knockouts are used [87]. Ploss et al. [88] used the slow glucose release of the growth system to mimic industrial carbon-limited growth conditions of *B. subtilis* to investigate the expression heterogeneity of secretory proteins with α-amylase AmyM from *Geobacillus stearothermophilus* as case example. Other applications of EnBase media even included the growth of lactobacilli [89, 90] and salmonella [91].

Furthermore, the newly developed growth systems, such as EnPresso, are widely compatible with a number of cultivation vessels from high-throughput small scale, over shake-flasks to larger reactors [48, 92–95], even in robotic automated set-ups [32, 96]. The scalability of such a system has been demonstrated for the production of different proteins by Glazyrina et al. [97], Li et al. [75] and Šiurkus et al. [22, 48, 98]. The latter is a great example for the successful implementation throughout different cultivation scales. The heterologous expression of RNase inhibitors is problematic because of product aggregation followed by low product yield. This was due to the unusual structure of this type of proteins characterized by a core of leucine-rich repeats in addition to 32 cysteins. To screen for the optimal expression system yielding soluble protein, the RNase inhibitor gene was cloned into a cytoplasmic expression vector library which contains 45 vectors differing in promoter strength, ribosomal binding sites and fusion tags. A luciferase based protein folding reporter system was used to monitor protein aggregation. The best conditions (maltose binding protein tag regardless promoter strength and ribosome binding sequence) were transferred to shake flask scale to establish the optimal substrate feeding rate. Since throughout the screening process EnBase provided fed-batch conditions the cultivations could be easily transferred to the bioreactor scale (fed-batch with glucose feed, using MSM medium). Cultivations in complex medium (LB medium) were done only for comparison. An important conclusion that we can draw from this study is that the amount of soluble and active product was instead of being influenced by promoter strength (i.e. induction strength) or cultivation temperature influenced by the specific growth rate prior to induction and also the medium composition. Differences between the scales did not appear because of the cultivation conditions, but rather because of lower oxygen transfer rates in small scale shaken cultures compared to the rates larger in bioreactors. The study Glazyrina et al. [97] comes to a very similar conclusion. Here the production of an alcohol dehydrogenase (ADH) could be run under comparable conditions from mL- to 60 L-scale due to the use of a smart cultivation system. Their results showed that either fed-batch conditions or the conditions provided by the EnBase Technology^®^ could be used to achieve much higher cell densities (up to 22 g L^−1^ for the fed-batch and up to 13 g L^−1^ for the EnBase cultivations).

In general one can summarize that advanced growth systems lead to higher protein yield; on average the EnBase technology enables at least fivefold higher protein yield than the traditional LB.

Finally, in the area of pharmaceutical protein production not only the host system plays a role, but also the media composition. Mainly mineral media are used. Especially when producing proteins in minimal medium for subsequent structural characterization by NMR, poor growth, low levels of expression, and insufficient labeled protein are very often problematic [99]. The application of an intelligent growth system can solve this problem by addition of a ^15^N labelled ammonium salt [100, 101]. Another example comes from scientists from Vernalis plc (UK), who were able to achieve an increase in productivity from 2.5 to 160 mg L^−1^ of a ^15^N-labeled protein [102].

### Outlook

During the last decade there have been significant advances in the heterologous expression of target proteins in bacterial (in particular *E. coli*), insect and mammalian cell cultures. These developments lead to a steady increase in the yield of recombinantly expressed proteins of high quality, in shorter time periods, and an overall higher success rate for expression of functional, active proteins used in downstream applications. Nevertheless, the constantly rising need for the production in therapeutics and large amounts of protein for biophysical and structural analysis does still require the development of novel expression technologies in the future, especially, to avoid/reduce the eminent problem of protein aggregation. Recently, this problem has been addressed from a different angle, including also the cell physiology and cultivation conditions as part of the solution.

It can be concluded that the application of smart media to obtain a controlled and balanced process is important to improve protein production. Summarizing, all the benefits of combining smart growth systems with conventional approaches and the wide applicability in terms of vessel-type, strains and production target should be universally adapted as a routine growth system in the everyday lab.

## Abbreviations

*E. coli*: *Escherichia coli*
Fab: fragment antigen binding
kDa: kilo dalton
LB: Luria–Bertani medium
NAD +/H: nicotinamide adenine dinucleotide
OD: optical density
TB: Terrific Broth medium
